# Pre-asthma: a useful concept? A EUFOREA paper. Part 2—late onset eosinophilic asthma

**DOI:** 10.3389/falgy.2024.1404735

**Published:** 2024-05-15

**Authors:** G. K. Scadding, C. Gray, D. M. Conti, M. McDonald, V. Backer, G. Scadding, M. Bernal-Sprekelsen, E. De Corso, Z. Diamant, C. Hopkins, M. Jesenak, P. Johansen, J. Kappen, J. Mullol, D. Price, S. Quirce, S. Reitsma, S. Toppila-Salmi, B. Senior, J. P. Thyssen, U. Wahn, P. W. Hellings

**Affiliations:** ^1^ Department of Allergy & Rhinology, Royal National ENT Hospital, London, United Kingdom; ^2^ Division of Immunity and Infection, University College, London, United Kingdom; ^3^ Paediatric Allergist, Red Cross Children's Hospital and University of Cape Town, Cape Town, South Africa; ^4^ Kidsallergy Centre, Cape Town, South Africa; ^5^ The European Forum for Research and Education in Allergy and Airway Diseases Scientific Expert Team Members, Brussels, Belgium; ^6^ Escuela de Doctorado UAM, Centro de Estudios de Posgrado, Universidad Autónoma de Madrid, Calle Francisco Tomás y Valiente, no 2, Ciudad Universitaria de Cantoblanco, Madrid, Spain; ^7^ The Allergy Clinic, Blairgowrie, Randburg, South Africa; ^8^ Department of Otorhinolaryngology, Head & Neck Surgery, and Audiology, Rigshospitalet, Copenhagen University, Copenhagen, Denmark; ^9^ Allergy, Royal Brompton Hospital, London, United Kingdom; ^10^ Otolaryngology-Department, Clinic Barcelona, Barcelona, Spain; ^11^ Otolaryngology-Department, University of Barcelona, Barcelona, Spain; ^12^ Otolaryngology Head and Neck Surgery, A. Gemelli University Hospital Foundation IRCCS, Rome, Italy; ^13^ Department of Respiratory Medicine & Allergology, Institute for Clinical Science, Skane University Hospital, Lund University, Lund, Sweden; ^14^ Department of Respiratory Medicine, First Faculty of Medicine, Charles University and Thomayer Hospital, Prague, Czech Republic; ^15^ Department Clinical Pharmacy and Pharmacology, University of Groningen, University Medical Center Groningen, Groningen, Netherlands; ^16^ Department of Microbiology Immunology & Transplantation, KU Leuven, Catholic University of Leuven, Leuven, Belgium; ^17^ Department of Rhinology and Skull Base Surgery, Guy’s and St Thomas’ Hospital NHS Foundation Trust, London, United Kingdom; ^18^ Department of Clinical Immunology and Allergology, University Teaching Hospital in Martin, Martin, Slovak Republic; ^19^ Department of Paediatrics, Jessenius Faculty of Medicine in Martin, Comenius University in Bratislava, University Teaching Hospital in Martin, Martin, Slovak Republic; ^20^ Department of Pulmonology and Phthisiology, Jessenius Faculty of Medicine in Martin, Comenius University in Bratislava, University Teaching Hospital in Martin, Martin, Slovak Republic; ^21^ Department of Dermatology, University of Zurich, Zurich, Switzerland; ^22^ Department of Dermatology, University Hospital of Zurich, Zurich, Switzerland; ^23^ Department of Pulmonology, STZ Centre of Excellence for Asthma, COPD and Respiratory Allergy, Franciscus Gasthuis & Vlietland, Rotterdam, Netherlands; ^24^ Rhinology Unit and Smell Clinic, ENT Department, Hospital Clínic, FRCB-IDIBAPS, Universitat de Barcelona, CIBERES, Barcelona, Spain; ^25^ Observational and Pragmatic Research Institute, Singapore, Singapore; ^26^ Centre of Academic Primary Care, Division of Applied Health Sciences, University of Aberdeen, Aberdeen, United Kingdom; ^27^ Department of Allergy, La Paz University Hospital, IdiPAZ, Madrid, Spain; ^28^ Department of Otorhinolarynogology and Head/Neck Surgery, Amsterdam University Medical Centres, University of Amsterdam, Amsterdam, Netherlands; ^29^ Department of Otorhinolaryngology, Kuopio University Hospital and University of Eastern Finland, Kuopio, Finland; ^30^ Department of Allergy, Inflammation Center, Helsinki University Hospital and University of Helsinki, Helsinki, Finland; ^31^ Department of Otolaryngology/Head and Neck Surgery, University of North Carolina at Chapel Hill, Chapel Hill, NC, United States; ^32^ Department of Dermatology, Bispebjerg Hospital, University of Copenhagen, Copenhagen, Denmark; ^33^ Department for Pediatric Pneumology and Immunology, Charite University Medicine, Berlin, Germany; ^34^ Department of Otorhinolaryngology-Head and Neck Surgery, University Hospitals, Leuven, Belgium; ^35^ Laboratory of Allergy and Clinical Immunology, University Hospitals Leuven, Leuven, Belgium; ^36^ Upper Airways Research Laboratory, Department of Head and Skin, Ghent University, Ghent, Belgium

**Keywords:** late onset asthma, non-allergic rhinitis, chronic rhinosinusitis with nasal polyps, eosinophils, mast cells, virulence genes, *S. aureus* biofilm

## Abstract

The concept of pre-diabetes has led to provision of measures to reduce disease progression through identification of subjects at risk of diabetes. We previously considered the idea of pre-asthma in relation to allergic asthma and considered that, in addition to the need to improve population health via multiple measures, including reduction of exposure to allergens and pollutants and avoidance of obesity, there are several possible specific means to reduce asthma development in those most at risk (pre- asthma). The most obvious is allergen immunotherapy (AIT), which when given for allergic rhinitis (AR) has reasonable evidence to support asthma prevention in children (2) but also needs further study as primary prevention. In this second paper we explore the possibilities for similar actions in late onset eosinophilic asthma.

## 1 Introduction

The concept of pre-diabetes has led to provision of measures to reduce disease progression through identification of subjects at risk of diabetes (1). We previously considered the idea of pre-asthma in relation to allergic asthma (2) (Table 1) and considered that, in addition to the need to improve population health via multiple measures, including reduction of exposure to allergens and pollutants and avoidance of obesity, there are several possible specific means to reduce asthma development in those most at risk (pre- asthma). The most obvious is allergen immunotherapy (AIT), which when given for allergic rhinitis (AR) has reasonable evidence to support asthma prevention in children (2) but also needs further study as primary prevention (4).

**Table 1:** Pre-asthma: allergic and late onset compared.

Pre-asthma	
Allergic asthma	Late onset asthma
Eosinophilic/T2/systemic IgE	Eosinophilic/T2/? local IgE
Higher incidence and prevalence in children. Higher morbidity and mortality in adults	Occurs more frequently in females and non-atopic patients
Typically begins in childhood as part of a collection of atopic disorders mediated by IgE	Typically developed in the adulthood by inflammatory mechanisms other than those induced by Th2 via IgE
High prevalence of upper airway disease	
Both genetic predisposition and environmental factors and triggers implicated	
Increased morbidity, more severe asthma symptoms, accelerated decline in lung function, reduced lung growth, an altered inflammatory phenotype, and reduced corticosteroid responsiveness with increasing neutrophilic inflammation in case of smokers (3).	
Exposure to irritants/pollutants contributes to worsening of symptoms	
Obesity could predispose to or worsen asthma. It is also associated with co-morbidities which can further worsen the clinical manifestations of asthma.	
Prevention from early ages (maternal, perinatal and postnatal measures)	Later preventive measures possible?
Progression of asthma from AR	Progression of asthma from NAR/CRSwNP

In this second paper we explore the possibilities for similar actions in late onset eosinophilic asthma. 
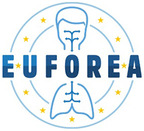


## 2 Late onset eosinophilic type 2 asthma

### 2.1 Pathophysiology

**Figure 1:**
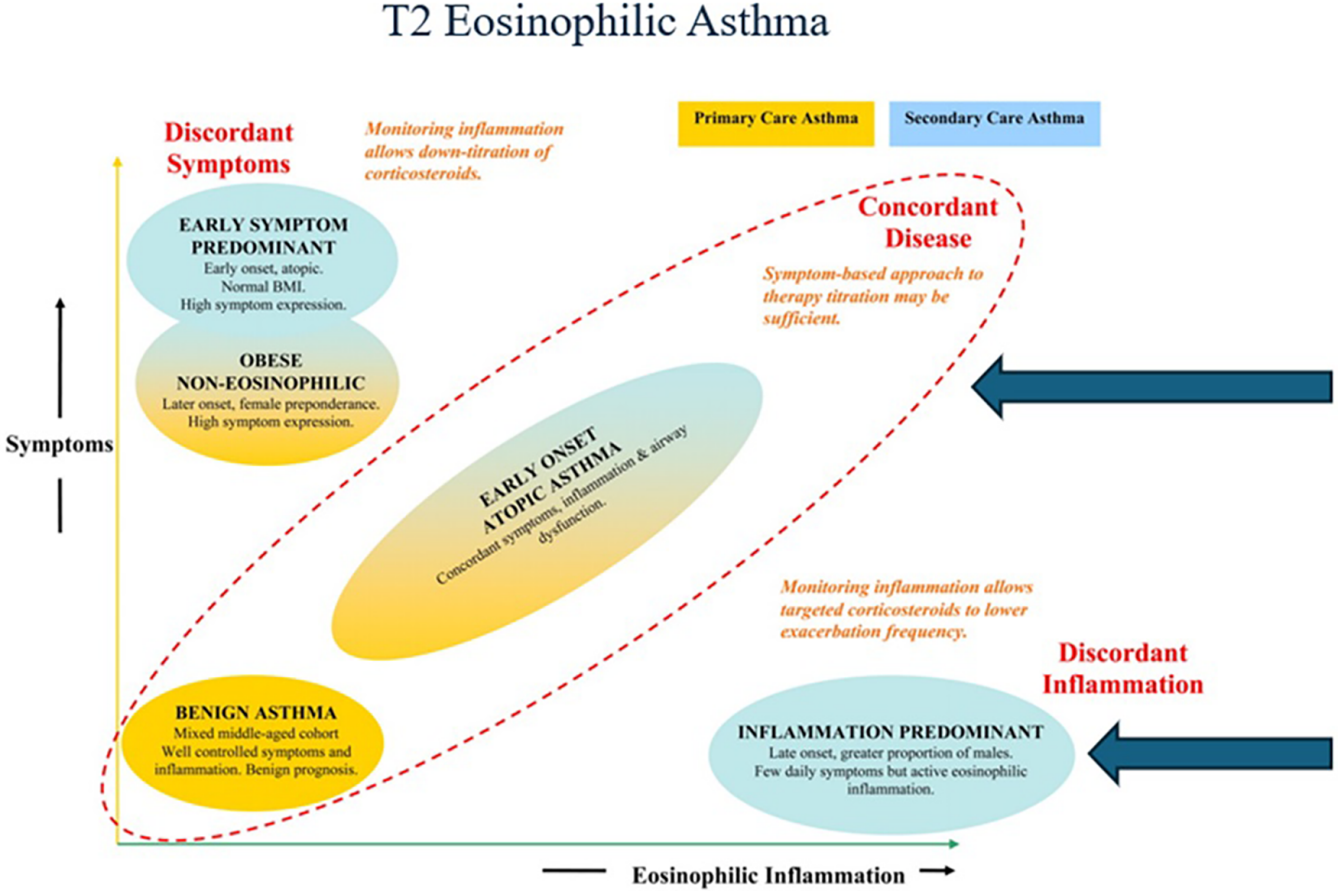
T2 eosinophilic asthma of late onset can be allergic or non—allergic as seen here in the asthma clusters described by Haldar et al. Adapted from Haldar et al. (6).

Inflammatory mechanisms other than those induced by Th2 via IgE may be involved in the pathophysiology of late onset asthma which has multiple phenotypes (5) (Figure 1). Indeed, the proportion of such asthma attributable to atopy is usually less than 50% (7). Whilst non-eosinophilic asthma remains relatively under-explored (8), there is now more and better characterisation of eosinophilic asthma, which lacks systemic IgE, i.e., asthma with low levels of serum IgE and negative skin prick tests to common allergens. Asthma with a type 2 (T2) endotype is driven by alarmins, IL-25, IL-33, and thymic stromal lymphopoetin (TSLP) either through naïve T-cells or through ILC2 cells, and involves the cytokines IL-4, IL-5 and IL-13, as well as eosinophils and, sometimes, IgE (9).

As in nasal polyps, IgE may present locally, rather than systemically (10). Circumstantial evidence that both atopic and nonatopic asthma may be mediated by local IgE in human bronchial mucosa (11). Although late-onset eosinophilic asthma has traditionally been considered non-allergic in nature, it can be associated with allergic features and comorbidities (12). In some patients, there is evidence that inflammation is driven by toxins from Staphylococcus aureus (vide infra). The possibility that late onset asthma is partly an auto-immune disorder has also been suggested, with some patients having pathogenic sputum autoantibodies against autologous eosinophil proteins (e.g., eosinophil peroxidase) (13, 14).

### 2.2 Predisposing conditions

The T2 form of asthma occurs more frequently in females and non-atopic patients and, like allergic asthma, and it may also be preceded by nasal disease, e.g., predominantly non-allergic rhinitis and/ or chronic rhinosinusitis with nasal polyposis (CRSwNP) (15).

#### 2.2.1 Non-allergic rhinitis (NAR)

NAR is defined as a chronic condition of the nasal mucosa that is not caused by allergy nor by an infectious agent, and subdivided into several groups, including gustatory, hormonal, drug-induced or idiopathic rhinitis. Typically, symptoms are triggered by irritants such as cigarette smoke, pollution, strong odors, physical exercise and/or changes in temperature and humidity. This induction of nasal symptoms by non-specific stimuli is called nasal hyperreactivity (NHR) and found in two thirds of patients with inflammatory conditions of the nasal and paranasal mucosa (16).

While allergic rhinitis is a well-established risk factor for asthma, the relationship between NAR and asthma is less clear. In the European Respiratory Health Survey NAR was associated with a nearly threefold risk of asthma development compared to subjects without rhinitis or atopy, only slightly lower than the risk for those young adults with allergic rhinitis (17). There is some evidence that individuals who have nasal symptoms of congestion and postnasal drip are most at risk (16). Another study found that NAR was associated with an increased risk of asthma exacerbations in individuals with comorbid asthma (18). Other studies have failed to confirm the link between NAR and comorbid asthma or have suggested that local allergic rhinitis has been misdiagnosed as non- allergic (19).

The relationship may be more complex and depend on factors such as the type and severity of NAR, which includes both inflammatory and neurogenic forms of rhinitis, as well as individual genetic and environmental factors. It is likely that inflammatory eosinophilic NAR, also called NARES, is the form of NAR, which is most associated with asthma development (20). Concomitant mast cells together with eosinophils in the nasal smears gave a particularly high risk for associated asthma (21).

#### 2.2.2 Chronic rhinosinusitis

Chronic Rhinosinusitis with Nasal Polyps (CRSwNP) is an inflammatory process affecting the lining of the nasal passages and sinuses. Individuals with CRSwNP are more likely to develop/have asthma than patients without polyposis (CRSsNP) (22) and are more likely to have severe disease (23). The prevalence of asthma in individuals with CRSwNP is estimated to be between 20% and 50% (24). The chronic inflammation associated with nasal polyps is variable, but those subjects with Type 2 eosinophilic polyps are highly likely to have concomitant asthma (25), particularly patients with allergic fungal rhinosinusitis (AFS) (26) or aspirin sensitivity (AERD). By extension, the proportion of comorbid asthma in patients with severe uncontrolled CRSwNP is very high and reaches up to ∼80% when biological therapy is indicated (27–29).

Indeed, approximately 20% of late onset asthmatics develop AERD or hypersensitivity to aspirin and other cyclooxygenase inhibitors (NSAID) (30). In a European study, most of the AERD patients developed upper airway symptoms prior to asthma (31). However, in an American study, with a majority of self-identifying, black study participants, the development of A-ERD was highly variable in onset and progression (32). Asthma occurred first in 50% of all participants, mainly in younger, female obese subjects. The “NSAID-sensitivity first” group was predominantly male odd's ratio (OR = 3.3), 95% confidence interval (CI) 1.5–7.4, *p* = 0.004) with exposure to pollutants, (OR = 4.4, CI 1.6–11.9, *p* = 0.003). A-ERD has evidence of additional eicosanoid dysregulation (33).

In a prospective study Sinus inflammation and chronic rhinosinusitis (CRS) [identified in two ways: validated text algorithm applied to sinus computerized tomography (CT) scan or two diagnoses] have been reported to be associated with a diagnosis of new-onset asthma in the following year (34).

## 3 Other risk factors for late onset T2 eosinophilic asthma

As in allergic asthma, there is evidence for both genetic and environmental influences (Box 1). 
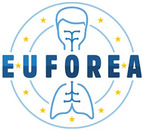

BOX 1 **Risk factors for late onset eosinophilic asthma.**

**Table:** 

Risk factors for late onset T2 eosinophilic asthma
A. Predisposing conditions
Female sex Upper airway disease Rhinitis—allergic and non—allergic Rhinosinusitis—CRSwNP
B. Other risk factors
Genes Familial aggregation of NP in CRS patients HLA-DPB1}in AERD ALOX-15}protects from AERD Environmental factors Allergen exposure Occupational exposure Tobacco smoke exposure Microbiome Staphylococcus aureus Obesity Hormonal factors Oral disease Psychosocial factors Sensitivity to aspirin/NSAIDS

Abbreviations missing (CRSwNP, chronic rhinosinusitis with nasal polyps; NP, nasal polyposis; CRS, chronic rhinosinusitis; AERD, aspirin-exacerbated respiratory disease; NSAIDS, non-steroidal anti-inflammatory drugs).

### 3.1 Genes

#### 3.1.1 Familial aggregation of NP in CRS patients

Familial aggregation of NP is demonstrated in CRS patients and correlates with disease severity (35, 36). A systematic review of all published data on genetic and epigenetic variations in CRSwNP since 2,000 identified over 150 genetic variants in 99 genes involved in pathogenesis. These were clustered into 8 main networks, linking genes involved in inflammation and immune response (e.g., MHC), cytokine genes (e.g., TNF), leukotriene metabolism, and the extracellular matrix. Eighty-nine miRNAs were also identified, associated mainly with the cell cycle, inflammation, and the immune response (37).

Genes related to Epithelial abnormalities, including filaggrin, were identified using whole exome sequencing in a small study (38).

#### 3.1.2 HLA-DPB1

AERD has several genetic associations in an asthmatic population with HLA-DPB1 gene polymorphism the most susceptible factor for the risk of AERD (39). Multiple other genes identified as possibly relevant in AERD are noted in Dahlin et al. (40).

#### 3.1.3 ALOX-15

Polyps from CRSwNP patients with AERD show elevated ALOX-15 expression, worse sinonasal disease and more operations (41) compared to patients without AERD.

The ALOX-15 mRNA expression level could distinguish between eosinophilic and non-eosinophilic CRSwNP, being significantly higher in eosinophilic ones (42).

Protection against AERD occurs with a loss of function mutation of ALOX-15 (43).

A promoter polymorphism enhances IgE responses to staphylococcal superantigens in adult asthmatics (44).

### 3.2 Environmental factors

#### 3.2.1 Allergen exposure

Indoor mould exposure in the last year, especially involving Cladosporium species, was associated with asthma symptoms and bronchial responsiveness (OR range, 1.14–1.44) (45).

Sensitization to perennial aeroallergens was present in 68% of adults with severe asthma, many of whom were likely late onset asthmatics. The most prevalent sensitizations were to *Dermatophagoides pteronyssinus, D. farinae, D. microceras, Aspergillus fumigatus, Staphylococcus aureus Toxic Shock Syndrome Toxin (TSST),* and *Candida albicans* (46).

#### 3.2.2 Occupational exposure

Exposure to certain substances in the workplace, such as irritants (47), chemicals, dust and fumes, can trigger asthma symptoms in adult life (48). The CONSTANCES cohort has shown that exposure to solvents and to irritants can trigger adult asthma (49, 50).

#### 3.2.3 Exposure to environmental tobacco smoke

Smoking is a risk factor for eosinophilic asthma in adult life (51). The effects of smoking on the risks of atopic and non-atopic asthma differ and are modified by gender. In women, but no in men, the risk of atopic asthma was increased (adjusted OR 1.24, 95% CI 0.83–1.85) by smoking. Recent smoking cessation was related to increased risk of both atopic (aOR 4.91, CI2.26–10.65) and non-atopic (aOR 4.37, CI 1.87–10.21) asthma. Ceasing to smoke over 12 months ago was related to increased risk of non-atopic asthma (aOR 1.57, CI 1.08–2.28), mainly in men (aOR 2.03, CI 1.06–3.88) (52).

#### 3.2.4 Microbiome

Microbial imbalance could be involved in the pathogenesis of upper and lower airway diseases, including asthma. The composition of the airway microbiome is susceptible to influences such as genetics, environmental exposures and medications. CRSwNP patient had reduced *Corynebacterium* and *Dolosigranulum* in their nasal samples compared to healthy controls. Bacterial genera such as *Lactobacillus, Escherichia coli, Shigella, Turicibacter, Clostridium, Enterococcus, and Romboutsia* were positively correlated with the severity of CRSwNP (53).

Viruses, rather than bacteria, constitute the largest proportion of the human microbiota. The lung virome has as yet been little studied. There is evidence of overabundance of cytomegalovirus (CMV) and Epstein Barr virus (EBV) in patients with asthma exacerbations, plus correlation with higher asthma severity, lower lung function and ACT scores. Conversely bacteriophage abundant in healthy controls was reduced in asthma, proportionally to severity (54).

##### 3.2.4.1 Staphylococcus. aureus

*S. aureus*-Serum IgE specific to *S. aureus* enterotoxin (SA-IgE) has been linked to adult-onset asthma and to more severe asthma (55–57). Nasal *S. aureus* carriage was positively associated with asthma prevalence in meta-analysis of five cross-sectional studies (OR 1.19, 95% CI 1.06–1.34) in the general adult population. It was positively associated with asthma in another meta-analysis of 11 studies of CRS patients (OR 1.86, CI 1.18–2.95) (58).

Much stronger associations exist with asthma prevalence for *S. aureus* recovery from surgical tissue specimens from CRS patients (OR 40.4, CI 10.5–155) than for *S. aureus* recovery from swab samples (OR 1.21, CI 0.99–1.48) (57). CRSwNP–derived *S. aureus* biofilms showed thicker biomass, higher colony-forming units, and higher exoprotein production than those from controls (*P* < 0.05). CRS severity scores were positively correlated with *S. aureus* biofilm properties and numbers of inflammatory cells (59).

More recently correlation of CD3 + cell subsets in the sinonasal tissue of CRS and non-CRS control patients with CRS severity scores, *in vitro*-grown biofilm properties and virulence genes of the corresponding patient-derived *S. aureus* have been demonstrated. In tissue harbouring the Staphylococcal isolates carrying the lukFPV gene (Panton–Valentine Leukocidin, a leukotoxin which lyses cells of the leukocytic lineage and destroys neutrophils) CD4T cell counts were higher (60).

Staphylococcal superantigens, such as enterotoxins (SEA), are highly mitogenic and stimulate activity in many T lymphocytes, leading to substantial mediator and proinflammatory cytokine release (61, 62), intensifying the Th2 response in the tissue and diminishing the immunosuppressive activity of Tregs (63).

#### 3.2.5 Obesity

A meta-analysis of several prospective studies involving more than 300,000 adults found a weight-response relationship between obesity and asthma. The risk of asthma in the overweight and in the obese groups compared with the lean group were OR 1.5 and OR 1.9, respectively (64, 65).

Arismendi and colleagues (65) note that obesity is a major modifiable risk factor for asthma, possibly acting via systemic inflammation, lung function alterations, metabolic dysregulation, microbiome changes, and epigenetic/genomic regulation. Adipose tissue is metabolically active, releasing pro-inflammatory cytokines, including leptin which induces the proliferation and survival of type 2 innate lymphoid cells (ILC2) and T helper 2 (Th2) cells, and also induces monocyte, CD4 + and CD8+ *T* cell activation.

Obese asthma patients are more likely to have a poor response to glucocorticoids. Vitamin D, which increases glucocorticoid effectiveness (66) is often low in obese subjects.

Several phenotypes of obesity-associated asthma exist. Patients with an earlier onset are likely to have T2 inflammation with more severe disease, while others may have a non-inflammatory form of asthma or neutrophilic disease (67–70).

#### 3.2.6 Hormonal factors

Oral contraceptives may be associated with asthma development (71) whereas hormone replacement therapy was associated with a reduced risk of development of late onset asthma in menopausal women (72).

#### 3.2.7 Oral disease

Adult asthmatics experience a higher risk for a major oral disease or oral-manifesting disease, but it is uncertain whether this is post or propter hoc (73).

#### 3.2.8 Psychosocial factors

Stress, depression and traumatic events in childhood have been reported as risk factors for adult-onset asthma, but there may be reverse causation (74).

#### 3.2.9 Poor quality sleep

The UK Biobank cohort involving 455,405 participants aged 38–73 years was employed in a large-scale prospective study looking at genes and sleep scores. Over the 10 years plus of follow-up 17.836 of these individuals were diagnosed with asthma. The hazard ratio (HR) for poor sleep compared to the low-risk group was 1.55 (95% CI: 1.45–1.65). Poor sleep was additive to high genetic susceptibility (HR (95% CI): 2.22 (1.97–2.49), *p* < 0.001) compared with the low-risk combination. Healthy sleep lowered the risk of asthma in all genetic susceptibility groups, HR being 0.63 (0.57–0.70) in the high-risk group. Risk analysis suggests that 19% of asthma cases could be prevented by improved sleep traits (75).

## 4 Possibilities for prevention

### 4.1 Allergen/occupational allergen exposure

Reduction of mould and house dust mites in homes by cleaning and proper ventilation should be encouraged. Wearing a face mask whilst cleaning might also be protective.

Proper precautions against exposure in relevant industries need to be enforced (76). Early identification of susceptible individuals (some of whom will initially develop rhinitis) by continued monitoring is advisable, with removal from subsequent exposure before irreversible asthma develops.

#### 4.1.1 Possible role of allergen immunotherapy

Allergen immunotherapy in adults with allergic rhinitis may present a potential pathway to reduction to progression to asthma. Although the effect appears stronger in children (77), a retrospective cohort study following up 332 non-asthmatic adults with allergic rhinitis over 9 years, showed that allergen immunotherapy significantly reduced the development of new onset asthma [OR 0.53, (0.32–0.86)] (78).

### 4.2 Smoking

Continued discouragement of initiation of smoking of all kinds is necessary, together with improved education about the underlying reasons behind this (79).

### 4.3 Obesity

Weight loss and vitamin D improve hyporesponsiveness to corticosteroids in obese asthma (80). Bariatric surgery improved corticosteroid responses of peripheral monocytes *in vitro* in a small study in obese asthmatics and normalized their adiponectin/leptin ratio and vitamin D levels (80). Following the breakthrough with semaglutide (marketed as Wegovy or Ozempic), other similar glucagon-like peptide (GLP-1) weight loss drugs are entering the market. There is evidence that these drugs may reduce diabetes and cardiovascular complications of obesity. Studies are needed to investigate if late onset asthma is also decreased by the use of GLP-1 drugs, which can reduce inflammation in the liver, kidneys, heart and brain. As for yet, there is no evidence of any effect of GLP-1 drugs on asthma. However, since immune cells do not express a high frequency of GLP-1 receptors, the drugs may have limited or no effect on asthma (81). Studies are needed.

### 4.4 Progression of asthma from NAR

Several prior studies have shown an association between AR and development of asthma, but only one small study found that intranasal corticosteroids reduced the incidence of asthma in non-allergic rhinitis (82). Further research is needed to confirm these findings.

### 4.5 Progression of asthma from CRSwNP

There is currently limited scientific evidence for asthma prevention in CRSwNP patients. Reduction in the burden of upper airway inflammation might be preventative. In CRSwNP, burden can be reduced both medically (appropriate medical treatment and biologics, in severe uncontrolled patients) and surgically (endoscopic sinus surgery), but in most patients it recurs over time, being ameliorated in the long term by regular intranasal corticosteroids (83, 84). It is conceivable that those CRSwNP patients, who are excellent responders to anti-leukotrienes, might show reduced progression to asthma (85, 86). To date, this association has not been investigated.

There is some evidence that early surgery of CRSwNP is associated with less progression to asthma (87, 88).

Debulking surgery in CRSwNP, removing extensive polyp tissue reduces asthma symptoms and decreases the release of LTE4, the major leukotriene metabolite in AERD and useful in diagnosis of AERD (89, 90). Better CRSwNP control leads to better asthma control (91). However, long-term sizeable observational studies are needed to see if early polyp surgery is associated with reduced progression to asthma. Retrospective data may be available from existing records.

#### 4.5.1 Possible role of aspirin desensitization

Patients with hypersensitivity to Non-Steroidal Anti-Inflammatory Drugs (NSAIDs), including aspirin, show a significant increased risk of asthma [OR 5.5 (4.84–6.26)] (92) and an increased risk of uncontrolled asthma (93).

In those with AERD presenting with nasal polyposis and/or chronic rhinosinusitis, aspirin desensitization may present a possible opportunity for reduction of progression to asthma or reduction in asthma severity. A meta-analysis of aspirin desensitization in AERD showed a trend towards improved lung function and asthma symptom and medications scores, but additional RCTs are needed to fully assess this (94).

#### 4.5.2 Effects of biologics

Monoclonal antibodies (mAbs) targeting IgE and cytokines implicated in the T2 inflammatory cascade of asthma have been developed (95–97).

These biological drugs include omalizumab (anti-IgE), mepolizumab and reslizumab (anti-IL-5), benralizumab (antagonist of the *α* subunit of IL-5 receptor), and dupilumab (directed against the *α* subunit of IL-4 receptor which is a common receptor for IL-4 and IL-13). Tezepelumab is an anti-alarmin directed against thymic stromal lymphopoietin (TSLP).

Clinical trials and real-world studies have shown their efficacy in T2 asthma in reducing exacerbations, improving asthma control, pulmonary function, and withdrawing or at least reducing oral corticosteroid use (OCS) (98–100).

There is also evidence of effective action in CRSwNP and in subjects with both CRSwNP and asthma (101), with greater efficacy in those with severe asthma (102). However, more research is needed to determine whether any biologic, if given to CRS patients, can prevent asthma development (97).

Since IL-5 and eosinophils predominate in CRSwNP, one would expect anti-IL-5 to be an effective treatment. However, more effective in CRSwNP is treatment with the molecule directed against the IL4R*α* (dupilumab) (103, 104). Dupilumab therapy can also reverse aspirin/NSAID sensitivity, protecting patients against unwonted drug-induced exacerbations (97, 105).

A registry of patients treated with biologics for CRSwNP needs to be established in order to investigate the effect of biologics on the progression of CRSwNP to asthma. EUFOREA is in the process of setting up such a data collection.

### 4.6 Microbiome

Chalermwatanachai and colleagues noted an abundance of *Phylum Proteobacteria* in patients with asthma—associated CRSwNP compared the those with CRSwNP alone in whom *Staphylococci* and *Moraxella* were less prevalent (53). Frequent use of antibiotics has led to increasing resistance rates, becoming now a major health concern. Novel therapeutic strategies, including anti-virulence treatments that directly or indirectly neutralize *S. aureus* toxins, are in development (106). In CRSwNP patients, *S. aureus* grows intramucosally and intracellularly in the polyp tissue (24). More than 600 proteins released by *S. aureus*, including virulence factors such as the enterotoxins, were identified in the upper airway mucosa of patients with CRSwNP by high-resolution mass spectrometry among these were (107).

Antibiotic therapy with macrolides over months improved CRSwNP and associated asthma symptoms in a small study (108). A larger macrolide study is ongoing (**EudraCT Number:** 2018-001100-11.) Doxycycline reduces polyp size in CRSwNP (109) and might also be preventative of asthma. Similarly Staphylococcal decolonization routines could be attempted in those with risk factors or asthma. Antibiotic use carries risks such as changes in nasal microbiome and antibiotic resistance (110).

A different approach, such as measures to disable toxin production (106), could prove preventative with fewer adverse effects, since Staphylococcal toxins appear relevant to disease causation. The use of highly bacteriophages to remove certain bacteria is under study (111).

### 4.7 Anti-oxidants

Oxidative stresses play a role in inflammatory airways diseases, and low antioxidant levels may be a risk factor for asthma inception (112). Diets deficient in anti-oxidant vitamins and minerals, including vitamins A, D, E, C as well as selenium and zinc, have been associated with increased asthma prevalence. Supplementation with such vitamins and minerals have shown promising results in some, but not all, studies and merit further study (113). Resveratrol, a plant derived bioactive polyphenol found naturally in red grapes, berries and certain nuts, and also commercially in supplements, has shown pleiotropic anti-inflammatory and anti-oxidative effects (114). Murine models have shown resveratrol to attenuate allergic asthma with potential therapeutic effects on respiratory system diseases (115). Studies supplementing resveratrol in patients at risk of asthma or with asthma are warranted.

### 4.8 Sleep medicine

Early detection and management of sleep disorders could be beneficial in reducing asthma incidence.

## 5 Discussion

Prevention of late onset eosinophilic asthma seems more challenging than prevention of allergic asthma, as many patients have no identifying atopic history, though some studies suggest the relevance of IgE, possibly on a local level (12, 116). General measures such as reduction of obesity plus careful checking for occupational or recreational exposures and sensitizations are relevant. As with allergic asthma, recent understanding of the role of highly processed foods in causing obesity would suggest that avoidance of such foods should be recommended. However, the issue of dietary healthy eating guidelines is sensible at a population level.

The existence of allergic fungal rhinosinusitis with small quantities of fungus in the sinuses causing extensive inflammation, high levels of IgE and associated asthma (allergic bronchopulmonary aspergillosis, ABPA) warrants a careful check for this in CRSwNP in relevant areas of the world, as surgical removal is a necessity. Mask-wearing in polluted areas plus saline nasal douching after exposure could conceivably reduce asthma incidence in those sensitive to moulds and fungi.

A family history of CRSwNP, particularly in the father, conveys a high risk for nasal polyp development (117). Similarly, a family history of asthma or aspirin/NSAID sensitivity increase the likelihood of AERD (38). In consequence, identification of pre-asthma, in which intermittent lower airways inflammation is occurring, would be possible using FeNO measurements in the offspring of such patients. Possible preventive measures could then include regular nasal douching to remove local contributory factors acting on the epithelium. This could be tried at the initial stages of non-allergic eosinophilic rhinitis, or at the first appearance of polyps. Supplemental treatment could be intranasal corticosteroids or a trial of inhibitors of staphylococcal toxin production, or their combination. Early polyp surgery improves the prognosis of CRSwNP (87) with fewer asthma subjects than in the late surgical group, so this should be encouraged. Subsequent regular therapy to reduce recurrence should again be intranasal corticosteroids and possibly a trial of inhibitors of staphylococcal toxin production. The use of monoclonal antibodies in CRSwNP should be recorded in databases so that these can be mined for useful data on asthma prevention and length of effectiveness.

There is a complex interplay between an individual's genetic predisposition and their exposure to various environmental triggers. This fact underscores the necessity of a holistic approach to prevention, considering both intrinsic and extrinsic factors. The possibilities for prevention extend beyond traditional interventions, suggesting innovative approaches such as the use of biologics targeting specific inflammatory pathways and the potential role of weight loss drugs in reducing the inflammatory burden associated with obesity, a known risk factor for asthma. The effect of healthy sleep, even in those of high genetic predisposition, is remarkable and deserves further prospective work.

The limitations and gaps in current knowledge, highlight the need for further research. This includes the need for large-scale observational studies to understand the long-term effectiveness of interventions like early surgery for CRSwNP and the use of monoclonal antibodies in preventing asthma development in patients with CRSwNP (Table 2). Upper airway disease, both AR and CRS, needs to be taken seriously. Since it is likely that effective AR and CRS treatment might prevent progression to asthma. Identification of some older subjects at risk of asthma followed by therapeutic intervention is now becoming a possibility.

**Table 2:** Need for future research on the development of late onset asthma.

Need for future research on the development of late onset asthma
**1. Large scale studies on Upper Airway disease** Retrospective- data mining- to determine who progresses from Upper Airway disease to asthma, from AR, NAR and CRS, effects of therapies used. Prospective- interventional- with pheno- and genotyping of subjects where possible- effects of therapies, pharmacological and biological (AIT and monoclonal antibodies) on progression to asthma -observational- maintenance of databases.
2. **Similar studies on obesity**: effects of diet, bariatric surgery and PG-1 inhibitors.
3. **Studies on the microbiome**—effects of interventions: diet, probiotics, antibiotics, toxin inhibitors, bacteriophages.
**4. Smoking:** Which messages work in preventing uptake of smoking/ vaping? Can use of these prevent asthma?
**5. Pollution:** Any discernible change in incidence of new asthma during lockdown? Are there benefits from traffic free zones? Does reduction of exposure to detergents, cleaning fluids reduce asthma incidence?
6. **Implications for oral contraceptive use:** Does age at first use, length of use, etc. matter?
7. **The relationship between asthma and oral disease:** Need integration of dental databases with medical ones, exploration of oral microbiome and asthma
8. **Psychological factors as cause or consequence**

In conclusion, this manuscript contributes significantly to the ongoing discourse on asthma prevention, offering valuable insights into the potential for reducing the incidence of late-onset eosinophilic asthma through a combination of early identification and management of precursor conditions, genetic and environmental risk factor modification, and innovative therapeutic interventions. The call for further research underscores the evolving nature of our understanding of asthma and the continuous quest for more effective preventive strategies.

## Data Availability

All relevant data is contained within the article: The original contributions presented in the study are included in the article, further inquiries can be directed to the corresponding author.
